# Battery deactivation with redox shuttles for safe and efficient recycling

**DOI:** 10.1038/s41598-024-53895-3

**Published:** 2024-02-11

**Authors:** Riho Mikita, Akitoshi Suzumura, Hiroki Kondo

**Affiliations:** Toyota Central R&D Laboratories, Inc., Nagakute, Aichi 480-1192 Japan

**Keywords:** Electrochemistry, Physical chemistry, Energy storage, Materials for energy and catalysis, Environmental chemistry, Sustainability

## Abstract

To safely recycle spent lithium-ion batteries (LIBs), their deactivation as a pretreatment is essential. However, the conventional deactivation methods, mainly inducing an external short circuit, cannot be applied to LIBs with disconnected electrical circuits or Li deposited, despite their safety risk. Here, we propose a deactivation method using redox shuttles (RSs). The addition of an RS with redox potentials located between the two electrode potentials into a LIB electrochemically induces an internal short circuit with or without disconnected electrical circuits. A fully charged LIB discharges to approximately 0 V when a deactivation agent containing ferrocene or phenothiazine as an RS is added. Moreover, we demonstrate that RSs introduced into LIB can simultaneously dissolve Li deposited on the negative electrode surface and return it to the positive electrode as mobile ions. These characteristics of our method contribute to the improvement in safety and collection rate of Li in the recycling processes, promoting the sustainability of LIBs.

## Introduction

With the recent increase in the adoption of electrification to reduce CO_2_ emission, lithium-ion batteries (LIBs) have become essential as energy-storage devices^1,2^. The substantial increase in the demand for LIBs has necessitated the development of efficient recycling systems for spent LIBs. Such systems will enable the sustainable utilization of rare materials and the reduction of the life cycle CO_2_ emission in battery technologies^3–5^. The existing technologies for recycling LIBs mainly include pyrometallurgy, hydrometallurgy, and direct recycling^6,7^. In the pyrometallurgical process, spent LIBs are generally smelted at high temperatures (> 1000 °C), following which the resulting alloys are separated into the component metals, such as Ni, Co, and Cu. In contrast, the hydrometallurgical process does not include the smelting step. Valuable metals are chemically collected from cell components separated based on their physical properties. In direct recycling, electrode-active materials collected from spent LIBs are reconditioned and reused without being separated into component metals. Among these processes, pyrometallurgy is accompanied by large amounts of CO_2_ emission, although it is simple and available on an industrial scale. Consequently, the approaches for recycling recently tend to be shifted toward the hydrometallurgical process or direct recycling.

In hydrometallurgical or direct recycling approaches, preventing safety risk is of paramount importance, especially in light of the recent increase in fires caused by improper treatment of spent LIBs^8,9^. These hazards primarily originate from the voltage or energy remaining in spent LIBs. When spent LIBs with residual voltage are crushed during recycling processes, they often experience temperature increases due to internal short circuit, leading to subsequent thermal runaway and toxic-gas generation. Furthermore, in the case of spent LIBs with Li deposition, the deposited Li increases the potential hazard because it can lower the thermal runaway temperature of LIBs or react vigorously with humidity when exposed to air^10–12^. Therefore, in hydrometallurgical or direct recycling processes, the initial step of discharging spent LIBs and deactivating the deposited Li is indispensable^5^. Currently, there are a few deactivation approaches for LIBs, including inducing an external short circuit using electronic loads^5^ or conductive liquids (e.g., aqueous solutions containing a salt, such as NaCl or Na_2_SO_4_)^13^. Among these methods, the salt-water-based method holds promise for application to increasingly diverse batteries due to its simplicity. However, this method is not without its drawbacks. For instance, the process involves the generation of H_2_ or Cl_2_ gas, or the leaching of Li or Cu components into salted water through the corrosion of the battery case, increasing safety risks in the pretreatment process and decreasing material collection rates^13,14^. Furthermore, this method requires post-treatment of the salted water used for battery deactivation. Consequently, the salt-water-based method proves challenging for the deactivation of the large number of spent batteries anticipated in the future. In contrast, deactivation through an external short circuit using electronic loads can solve these problems. However, it cannot discharge LIBs with disconnected electrical circuits due to the operation of safety devices such as a current interrupt device (CID) or deactivates deposited Li, which is often electrically disconnected from the electrode. If such LIBs are sent to the crushing step without being sufficiently deactivated, hazards may arise due to their residual voltage, as described above and reported in previous studies^9,15^. Currently, these LIBs can only be rendered safe through roasting, resulting in increased CO_2_ emissions and reduced recycling efficiency. In such situations, an alternative method that ensures reliable deactivation with minimal environmental impact and high safety is required.

**Figure 1:**
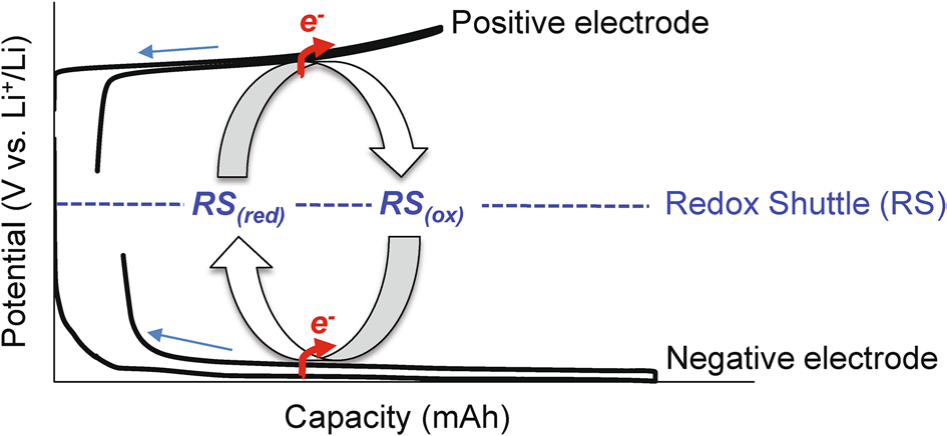
Proposed mechanism for the deactivation of spent LIBs using a redox shuttle (RS). RS_(red)_ and RS_(ox)_ represent the reduced and oxidized forms, respectively.

Herein, we propose a deactivation method using a redox shuttle (RS) to address the above problems in the conventional methods. RSs that undergo reversible redox reactions at a characteristic potential are conventionally employed as active materials in redox-flow batteries^16,17^, over-charge/over-discharge protection agents in LIBs^18,19^, or reconditioning agents for positive-electrode active materials^20–22^. Figure 1 illustrates the basic principle of our deactivation method using an RS. In our method, a small hole is created in a spent LIB, after which a solution containing RSs is introduced through the hole. When the redox potentials of an RS are located between the positive and negative electrode potentials, the positive electrode can be reduced with electron acceptance from the reduced form of the RS (RS_(red)_) and lithium acceptance from the electrolyte solution. Contrarily, the negative electrode can be oxidized with electron donation to the oxidized form of the RS (RS_(ox)_) and lithium donation to the electrolyte solution. Shuttling these redox events induces electron transfer from the negative electrode to the positive electrode, resulting in the deactivation of LIBs. In other words, the RS electrochemically induces an internal short circuit. Thus, this method can be theoretically employed to discharge a LIB with a disconnected electrical circuit or deactivate metallic Li deposited on the negative electrode surface in a spent LIB, which is difficult to achieve by the conventional methods inducing an external short circuit. Especially for the latter characteristics, the deposited Li becomes mobile ion and is returned to the positive electrode by our deactivation method. This contributes to the improvement in not only safety of recycling process itself, but also collection rate of Li from spent LIBs, which can reduce its resource depletion or cost increase potential in the future. In this paper, we demonstrate the application of RSs to deactivate LIBs with or without deposited Li and furthermore examine the relationship between the electrochemical properties of the RSs and their LIB deactivation ability to design deactivating agents for LIBs.

## Results and discussion

**Figure 2:**
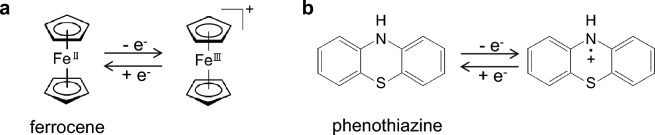
Redox reaction of (**a**) ferrocene and (**b**) phenothiazine.

Ferrocene and phenothiazine (Fig. 2) were employed as RSs for the deactivation of LIBs. The cyclic voltammetry (CV) measurements of ferrocene and phenothiazine in the lithium hexafluorophosphate (LiPF_6_) electrolyte solution were performed to evaluate their electrochemical properties in the typical LIB electrolyte solution containing LiPF_6_ (1.0 mol L^−1^) in a mixture of ethylene carbonate (EC), dimethyl carbonate (DMC), and ethyl methyl carbonate (EMC) (volume ratio = 30:40:30).

**Figure 3:**
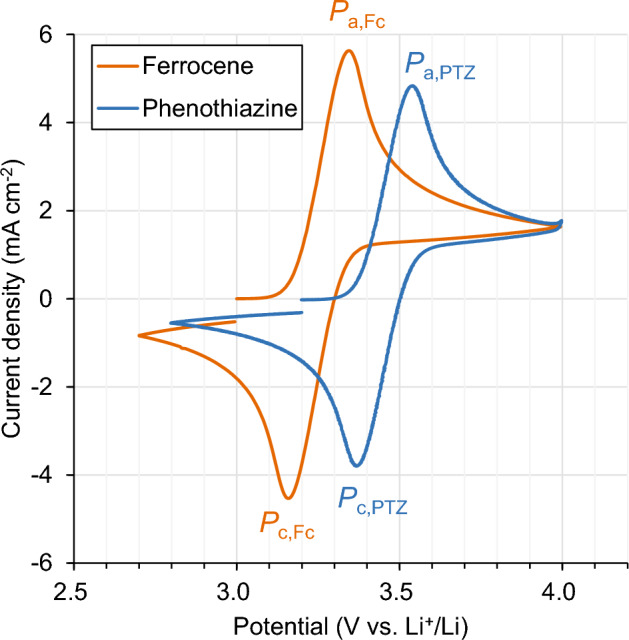
Cyclic voltammograms of ferrocene and phenothiazine with a supporting electrolyte of 1 M LiPF_6_ in a blend of ethylene carbonate (EC), dimethyl carbonate (DMC), and ethyl methyl carbonate (EMC) (volume ratio = 30:40:30), recorded on a glassy carbon electrode at a scan rate of 50 mV s^−1^ at 20 °C.

Figure 3 shows their cyclic voltammograms measured at a scan rate of 50 mV s^−1^ at 20 °C. In the cyclic voltammograms of ferrocene and phenothiazine, the positive potential scan from the open circuit potential (OCP) to 4.0 V *vs*. Li^+^/Li showed an anodic current peak indicated by *P*_a,Fc_ and *P*_a,PTZ_ in Fig. 3 at 3.34 V *vs*. Li^+^/Li and 3.54 V *vs*. Li^+^/Li, respectively. These peaks correspond to the oxidations of ferrocene and phenothiazine, respectively (Fig. 2). Afterward, when the potential scan direction was reversed at 4.0 V *vs*. Li^+^/Li, cathodic current peaks, *P*_c,Fc_ and *P*_c,PTZ_ were observed at 3.16 V *vs*. Li^+^/Li and 3.37 V *vs*. Li^+^/Li, respectively. These peaks, in turn, are ascribed to the reduction of the oxidized forms of ferrocene and phenothiazine. A formal redox potential, *E*, of the RS can be represented using its anodic and cathodic peak potentials (*E*_a_ and *E*_c_, respectively), as follows:

1 $$E=\frac{{E}_{a}+{E}_{c}}{2}.$$

From Eq. (1), the formal redox potentials of ferrocene and phenothiazine in the LiPF_6_ electrolyte solution were calculated to be 3.25 V *vs*. Li^+^/Li and 3.45 V *vs*. Li^+^/Li, respectively, which are comparable to those reported in other publications^23,24^.

Since the RSs added to the LIBs travel between the positive and negative electrodes to induce electron transfer, it is expected that the diffusion coefficients of the RSs in the electrolyte solution affect their LIB deactivation rates. Thus, to evaluate the diffusion coefficients of ferrocene and phenothiazine in the electrolyte solution, the scan-rate dependence of the cyclic voltammogram was measured at 20 °C (Figs. S1a and S2a). The cyclic voltammograms of ferrocene and phenothiazine show a similar dependence on the scan rates: the peak potential difference increases with the scan rate. In addition, as shown in Figs. S1b and S2b, the peak current shows an almost linear dependence on the square root of the scan rate, with the correlation coefficients close to 1. These results indicate that the charge-transfer reaction on the electrode surface is an irreversible process. In this case, the diffusion coefficients of the RSs in the electrolyte solution can be obtained using the following Randles–Sevcik equations for an irreversible redox system (Eqs. (2) and (3))^25^:

2 $$i_{p}^{a} = \left( {0.4958} \right)nFAD_{R}^{{\frac{1}{2}}} \left( {\frac{{\alpha _{a} n_{c} F}}{{RT}}} \right)^{{\frac{1}{2}}} C_{R}^{*} \nu ^{{\frac{1}{2}}}$$

3 $$i_{p}^{c} = \left( {0.4958} \right)nFAD_{0}^{{\frac{1}{2}}} \left( {\frac{{\alpha _{c} n_{a} F}}{{RT}}} \right)^{{\frac{1}{2}}} C_{0}^{*} \nu ^{{\frac{1}{2}}}$$

where superscripts or subscripts “*a*,” “*c*,” “*O*,” and “*R*” represent the anodic reaction, cathodic reaction, oxidized form of the RS, and reduced form of the RS, respectively. Parameters *i*_*p*_, *n*, *F*, *A*, *D*, *α*, *R*, *T*, C^*^, and *ν* represent the peak current in the cyclic voltammogram, the number of transferred electrons in the redox reaction, Faraday’s constant, electrode area, diffusion coefficient of the RS, electron-transfer coefficient, gas constant, temperature, concentration of the RS, and scan rate, respectively. From Eqs. (2) and (3), the absolute values of the slope of the fitting lines for the plot of the peak current (*i*_*p*_) versus the square root of the scan rate (*ν*^1/2^) give the diffusion coefficients (*D*) of the RSs. The parameters used in this study and obtained diffusion coefficients are listed in Tables S1 and S2. The diffusion coefficients of ferrocene and phenothiazine were calculated to be ca. 1.0 × 10^−10^ m^2^ s^−1^.

The property of the RSs as deactivating agents for the LIBs was investigated using pouch cells composed of a LiNi_1/3_Co_1/3_Mn_1/3_O_2_ (hereinafter abbreviated as NCM) positive electrode and a graphite negative electrode. Figure S3 shows the initial charge/discharge performance of each electrode and the pouch cell used for the present experiments. The cell discharge capacity from 4.1 V (i.e., SOC100%) to 3 V (i.e., SOC0%) is 17 mAh. Moreover, these charge/discharge profiles show an irreversible capacity loss, which can originate from the formation of the solid–electrolyte interphase (SEI) on the negative electrode. Figure S4 shows the voltage profile of the pouch cell when discharged to 0 V at a constant current. Considering the initial capacity loss of each electrode shown in Fig. S3, the cell voltage reaches 0 V, which is probably accompanied by an increase in the negative electrode potential to above 3 V *vs.* Li^+^/Li in the terminal stage of discharge. The RS dissolved in the mixture of EC, DMC, and EMC (volume ratio = 30:40:30) was added into the pouch cell fully charged up to 4.1 V.

**Figure 4:**
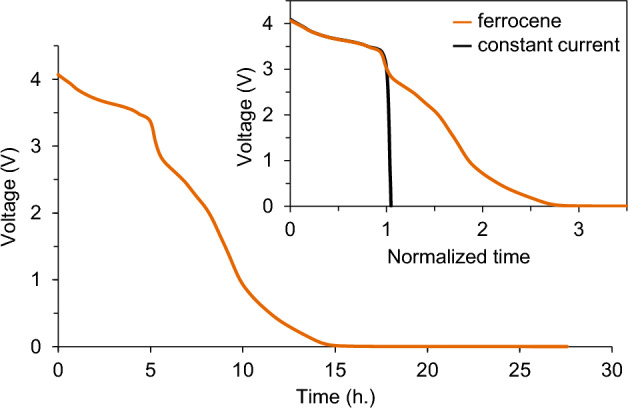
Discharge curves of the graphite/LiNi_1/3_Co_1/3_Mn_1/3_O_2_ cell with a solution of ferrocene. The inset shows the comparison of the discharge curves between discharging by ferrocene and applying a constant current. The horizontal axis is normalized by time at the voltage of 3 V.

Figure 4 shows the open circuit voltage (OCV) of the cell against time after the addition of the ferrocene solution. The OCV of the cell spontaneously decreased from 4.1 to 3 V in 5.5 h and subsequently to 0.1 V in 15 h. Since the added ferrocene corresponds to 10% of the cell capacity, the discharge of the cell is considered to occur via at least 10 redox cycles between the positive and negative electrodes. This result strongly indicates that ferrocene can work as a deactivating agent for LIB according to our proposed mechanism illustrated in Fig. 1. As shown in the inset of Fig. 4, the discharge profile with ferrocene shows similarity with the constant-current discharge curve (Fig. S4) in the voltage range of 3.0–4.1 V. Additionally, we plot the values obtained by differentiating the horizontal-axis value with respect to the vertical-axis value against the cell voltage. As shown in Fig. S5, the differential curve of time over the cell voltage (d*t*/d*V* curve) for the discharge curve with ferrocene shows three local minima at around 3.4 V, 3.6 V, and 3.9 V, similar to the differential curve of the cell capacity over the cell voltage (d*Q*/d*V* curve) for the constant-current discharge curve, which can be ascribed to the potential plateau in the lithiation process of NCM and the delithiation process of the graphite electrode^26^. Considering that the shape of the d*Q*/d*V* curve is similar to that of the d*t*/d*V* curve in the case of constant-current discharge, these similarities in the discharge profiles between the ferrocene-induced discharge and the constant-current discharge suggest that the discharge rates of the positive and negative electrodes with ferrocene are constant, although the positive electrode potential decreases with deactivation.

Here, we assume that the LIB discharge with an RS (Fig. 1) is divided into two processes: charge transfer at the electrode/electrolyte interface and RS diffusion between the positive and negative electrodes, as illustrated in Fig. S6. The charge-transfer current density (*i*_ct_) can depend on the overpotential (*η*) as well as the exchange current density (*i*_0_) as expressed in the following Butler–Volmer Eq. (4):

4 $${i}_{ct}={i}_{0}\left[{\text{exp}}\left(\frac{\alpha nF\eta }{RT}\right)-{\text{exp}}\left(-\frac{\left(1-\alpha \right)nF\eta }{RT}\right)\right].$$

In contrast, the diffusion current density (*i*_diff_) can be determined by the diffusion coefficient (*D*) and the concentration gradient ($\nabla c$) of the RSs, as expressed in Eq. (5):

5 $${i}_{diff}=nFD\nabla c.$$

When the cell is deactivated by the RSs, the observed current density can be expressed as a sum of *i*_ct_ and *i*_diff_, and the rate-determining step for the cell deactivation is determined by which one is dominant. For the charge-transfer-controlled reaction, the reaction rate should decrease with decreasing overpotential (*η*).

In the present result (Fig. 4), the discharge rate by ferrocene was almost constant in the voltage range of 3.0–4.1 V, despite the decrease in the overpotential for the oxidation of ferrocene on the positive electrode with discharging. This indicates that the charge-transfer reaction is fast enough that the concentration of ferrocene or its oxidized form at each electrode surface is almost zero and that their concentration gradient in the electrolyte reaches a steady state. Consequently, the constant discharge rate strongly indicates that the discharge reaction is diffusion-controlled. This result provides some insights into the design of deactivating agents for LIBs. The discharge of LIBs in this voltage range can be accelerated by increasing the diffusion coefficients of the RSs, e.g., by employing low-viscosity solvents.

In contrast, as shown in the inset of Fig. 4, the discharge rate with ferrocene decreased at voltages below 3.0 V, which can be related to the decrease in the overpotential on the positive electrode. Based on the constant-current potential profiles of NCM and the graphite electrodes (Fig. S3a), the positive electrode potential at the cell voltage of 3 V is expected to be approximately 3.6 V *vs*. Li^+^/Li, which is close to the redox potential of ferrocene (3.25 V *vs*. Li^+^/Li, Fig. 3). Therefore, the charge-transfer rate on the positive electrode decreases because of the decrease in the overpotential. In addition, the charge-transfer reaction rate on the negative electrode is possibly related to the decrease in the discharge rate at the cell voltage of 3 V. As shown in Fig. S3a, the negative electrode potential at this cell voltage is expected to be approximately 0.6 V *vs*. Li^+^/Li, where most of the Li ions in the graphite are already deintercalated. Thus, the anodic reaction of the graphite electrode can proceed via reactions other than delithiation (e.g., the decomposition of the solid electrolyte interphase^27,28^), although they are probably slower than delithiation. Consequently, for the addition of ferrocene as a deactivating agent into the present cell, the discharge rate decreases gradually as the overpotential decreases with discharging at voltages below 3 V.

The LIB deactivated with ferrocene was disassembled in an Ar atmosphere. The positive and negative electrode potentials after the deactivation were measured to be 3.25 V *vs*. Li^+^/Li and 3.23 V *vs*. Li^+^/Li, respectively, which are almost equal to the redox potential of ferrocene in the electrolyte solution (3.25 V *vs*. Li^+^/Li, Fig. 3). This result is consistent with the proposed mechanism of the deactivation with RSs, in which the discharge is driven by the potential difference between the electrodes and the RS, as illustrated in Fig. 1.

The discharge behavior of the LIB with phenothiazine was also investigated under the same condition. As shown in Fig. 5, the cell voltage decreased from 4.1 to 3 V in 38 h and subsequently to 0.1 V in 74 h after adding the solution of phenothiazine whose amount corresponds to 10% of the cell capacity. This suggests that phenothiazine solution also works as a deactivating agent for the LIBs. As shown in the inset of Fig. 5, the shape of the OCV curve in the voltage range of 3.4–4.1 V is similar to that of the constant-current discharge profile. Based on the above assumption that the discharge process with RSs can be divided into the charge-transfer step and diffusion step (Fig. S6), this similarity suggests that the discharge of the LIB with phenothiazine is diffusion-controlled in the voltage range of 3.4–4.1 V. Based on the constant-current potential profile of each electrode (Fig. S3a), at the cell voltage of 3.4 V, the positive electrode potential probably reaches approximately 3.7 V *vs*. Li^+^/Li, which is close to the redox potential of phenothiazine (3.45 V *vs*. Li^+^/Li, Fig. 3), whereas the negative electrode potential is expected to be ca. 0.3 V *vs*. Li^+^/Li. Here, comparing the OCV profiles between the discharge with ferrocene and phenothiazine (Fig. S7), we established that the cell voltage where the discharge rate decreased as the discharge becomes charge transfer-controlled is ca. 0.4 V higher for the discharge with phenothiazine than that with ferrocene. This implies that the decrease in the discharge rate at these cell voltages is mainly attributed to the decrease in the overpotential on the positive electrode, which can be affected by the redox potential of the added RSs.

**﻿Figure 5:**
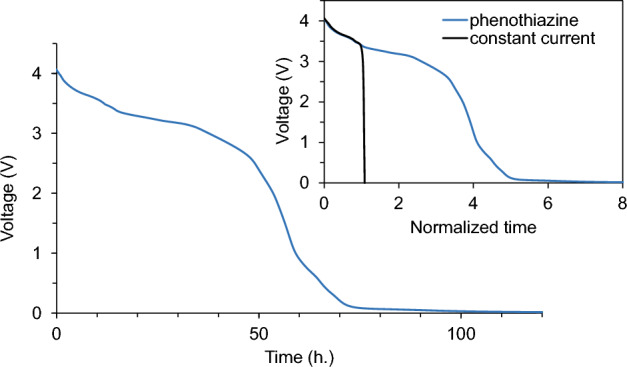
Discharge curves of the graphite/LiNi_1/3_Co_1/3_Mn_1/3_O_2_ cell with the solution of phenothiazine. The inset shows the comparison curve of the discharge between adding the phenothiazine solution and applying a constant current. The horizontal axis is normalized by time at a voltage of 3.4 V.

The cell deactivated with phenothiazine was also disassembled in an Ar-filled glove box. The potentials of the positive and negative electrodes were 3.3 V *vs*. Li^+^/Li and 3.2 V *vs*. Li^+^/Li, respectively, which are close to the redox potential of phenothiazine in the electrolyte solution. This indicates that the discharge of LIB is driven by the potential difference between the electrodes and phenothiazine, as shown in Fig. 1. Note that the slight difference between the electrode potentials after deactivation and the redox potential of phenothiazine obtained in the CV measurements (Fig. 3) can be attributed to the difference in the concentration of the supporting electrolyte.

Consequently, we demonstrated that ferrocene and phenothiazine act as deactivating agents for LIBs. Although, in the present experiments, a redox shuttle was introduced to the cell after only two cycles of charge and discharge to prove the concept, this method can also be applied to heavily cycled batteries with thick SEI films or reduced electrolyte amounts. Regarding the effect of SEI, various mechanisms of charge transfer between the negative electrode and the redox shuttle via SEI have been proposed so far^29^. The discharge rate may change depending on the composition or amount of SEI, but the battery discharge itself is achievable using RSs. Additionally, the redox shuttle is added in a dissolved form in the carbonate solvent, which serves as the electrolyte solvent. Therefore, this method can also discharge batteries in which the electrolyte dries up, provided the injected redox shuttle solution penetrates the electrodes or the separator.

Moreover, the composition of the redox shuttle, particularly the metal species, affects the separation step of the metal species in the subsequent recycling process. For example, when ferrocene is used to deactivate Li(Ni,Co,Mn)O_2_-based batteries, a new metal species (Fe) is introduced into the battery during the deactivation steps. Consequently, a new step for separately collecting Fe becomes necessary in the subsequent process, potentially increasing costs and decreasing separation efficiency. To address this concern, an organic redox shuttle, such as phenothiazine, is considered preferable to ferrocene. On the other hand, the rising demand for batteries using LiFePO_4_ as the positive electrode, driven by considerations of their durability and cost, suggests an impending need for recycling LiFePO_4_-based batteries. Given that LiFePO_4_-based batteries contain Fe, the use of ferrocene as a deactivating agent has minimal impact on the recycling process. Ferrocene, known for its stable redox performance, remains a viable choice for deactivating LiFePO_4_-based batteries. Additionally, the above experimental results using pouch cells indicate that these RSs induce an electrochemical internal short circuit in the LIBs, along with our proposed mechanism illustrated in Fig. 1. From this, one can expect that the RSs can also deactivate the Li deposited on the negative electrode surface, which is often electrically disconnected from the electrodes. Subsequently, we evaluated the deactivation performance of the LIB with Li deposited using phenothiazine as the deactivating agent.

**Figure 6:**
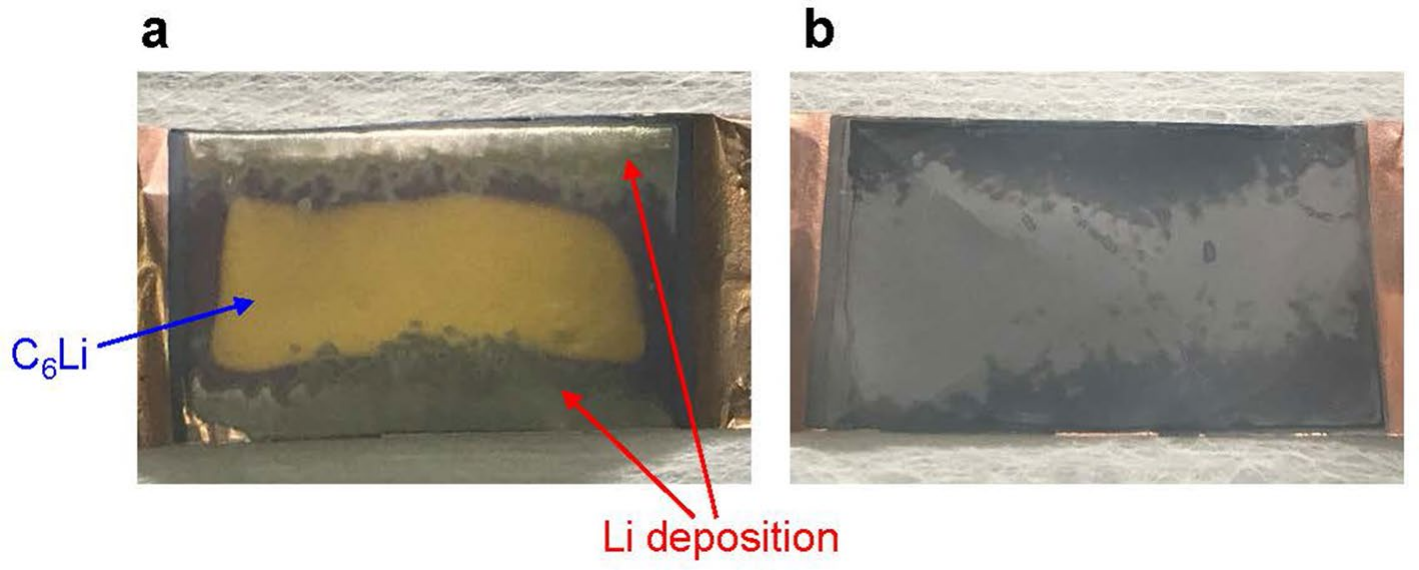
Images of the graphite negative electrode (**a**) after high-rate charge and discharge cycles and (**b**) after high-rate cycles and subsequent deactivation with phenothiazine.

We prepared two pouch cells with Li deposited by high-rate charge/discharge cycles under the same condition. As shown in Fig. S8, the discharge capacity of the two cells decreased through high-rate cycles, probably due to Li deposition. The degrees of the capacity decrease were similar, indicating that almost the same amounts of Li were deposited in these cells. After high-rate cycles, both cells were charged to SOC100%. Thereafter, one cell was disassembled without deactivation to examine the initial condition of the negative electrode surface, whereas the other cell was disassembled after deactivation with a phenothiazine solution. In the former cell, Li deposition was confirmed on the edge of the negative electrode surface, whereas gold-colored graphite, believed to be C_6_Li, was observed near the center, as shown in Fig. 6a. In contrast, after the addition of the phenothiazine solution, the cell voltage decreased from 4.1 to ca. 0 V (Fig. S9), and the Li on the edge of the electrode surface disappeared, whereas the color of the graphite near the center turned to gray from gold with discharging, as shown in Fig. 6b. As the amounts of Li deposited after high-rate cycles may be similar between these two cells, Li is considered to deactivate by the RS reaction of phenothiazine.

**Figure 7:**
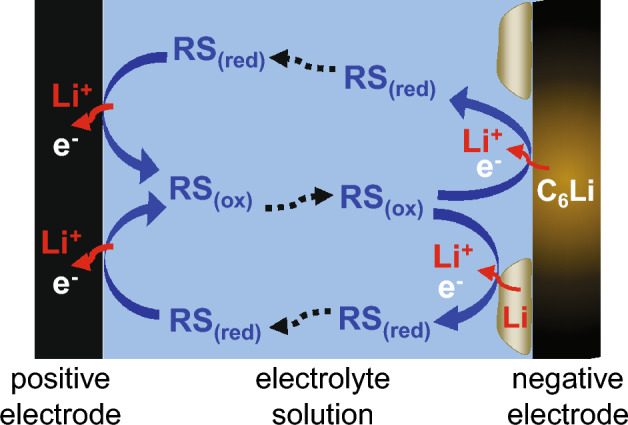
Mechanism of deactivating deposited Li using a redox shuttle (RS). RS_(red)_ and RS_(ox)_ denote the reduced and oxidized states of the RS, respectively.

A possible mechanism for the deactivation of deposited Li with the RS is illustrated in Fig. 7. The deposited Li becomes mobile ion by its oxidation (i.e., dissolution) accompanied by the electron donation to the oxidized form of the RS, and then the Li ion is returned to the positive electrode with electron acceptance from the reduced form of RS. This means that the deposited Li becomes collectible, which is conventionally difficult to achieve. These unique characteristics of our proposed deactivation method can not only improve the safety of the recycling processes, but also reduce the resource depletion or cost increase potential of Li in the future, contributing to the sustainability of LIBs.

## Summary

The present study demonstrated the ability of RSs to deactivate LIBs. The candidate RSs, ferrocene and phenothiazine, can deactivate LIBs using the potential difference with the electrodes as the driving force. The discharge reaction was diffusion-controlled at the initial stage, and the effects of the charge-transfer rate could not be ignored at the terminal stage mainly because of the decrease in the overpotential on the electrodes. These results suggest that factors determining the discharge rate depend on the discharge stage. The present findings provide insights for designing an RS solution for the deactivation of LIB according to the battery configuration or target-deactivation performance. Moreover, we experimentally confirmed that the RS-based pretreatment can return the Li species deposited on the negative electrode surface to the positive electrode as mobile ions, as well as deeply discharged the electrodes. Our RS-based method of electrochemically inducing an internal short circuit proves effective in deactivating batteries with disconnected electrical circuits or deposited Li—a challenge in conventional methods. This contributes to the improvement in the safety and collection rate of Li in the recycling processes. Our method is anticipated to serve as an alternative to conventional methods or an option for treating batteries that conventional methods struggle to address. However, future efforts should focus on developing a simplified method of adding redox shuttles or incorporating deactivating agents into battery design to ensure ease of implementation.

## Methods

### Preparation of redox shuttles

Commercially available reagents of ferrocene and phenothiazine (Tokyo Chemical Industry Co., Ltd.) were obtained and used as received.

### Cyclic voltammetry measurements

Cyclic voltammetry (CV) measurements were conducted to assess the electrochemical properties of the candidate RSs using H-type cells equipped with three electrodes: a glassy carbon electrode as the working electrode, and two Li electrodes serving as the counter and reference electrodes. The RS (0.05 mol L^−1^) was dissolved in a typical electrolyte solution containing LiPF_6_ (1.0 mol L^−1^) in a mixture of EC, DMC, and EMC (volume ratio = 30:40:30). As illustrated in Fig. S10, the counter and reference Li electrodes were separated from the RSs by a glass filter and a porous glass, respectively, to prevent any undesired reactions between the RS and the Li. All the solutions and the H-type cell were prepared in an Ar-filled glove box.

The potential of the glassy carbon working electrode relative to the reference Li electrode was scanned at a series of scan rates ranging from 1 to 1000 mV s^−1^, while maintaining a constant temperature of 20 °C. The specific potential scan range and direction were determined based on the redox potential and the redox state of the RSs. Given that ferrocene and phenothiazine represent the reduced forms (Fig. 2), the initial scan direction was set as positive. The potential scan range was defined as 2.7–4.0 V *vs*. Li^+^/Li for ferrocene and 2.8–4.0 V *vs*. Li^+^/Li for phenothiazine.

### Deactivation of lithium-ion batteries using redox shuttles

The ability of the RSs to deactivate LIBs was examined using pouch-type LIBs consisting of an NCM positive electrode and a graphite negative electrode. The NCM electrode was fabricated by coating a slurry of NCM (NCM-01ST-5P, Toda Kogyo Co., Ltd.), acetylene black (HS-100, Denka Co., Ltd.), and poly(vinylidene) fluoride (KF-7305, Kureha Co., Ltd.) in *N*-methyl-2-pyrrolidone (weight ratio = 93:4:3) onto an Al foil with a loading weight of ca. 12 mg cm^−2^. The graphite electrode was prepared by coating a slurry of spherical particles of graphite (OMAC1.0ss, Osaka Gas Chemical Company Co., Ltd.), scalelike graphite (SFG6, TIMCAL Co., Ltd.), carboxymethyl cellulose (CMC2200, Daicel Miraizu Co., Ltd.), and styrene butadiene rubber (TRD2001, JSR Co., Ltd.) in water (weight ratio = 93.1:4.9:1:1) onto a Cu foil with a loading weight of ca. 8.0 mg cm^−2^. The NCM and graphite electrodes were dried at 120 °C for 6 h under vacuum and subsequently pressed to densities equal to 2.6 g cm^−3^ and 1.3 g cm^−3^, respectively.

The electrochemical properties of each electrode were examined using coin-type cells composed of NCM or the graphite electrode with an area of ca. 2 cm^2^ (ϕ16 mm) as the working electrode, Li as the counter electrode, and a polyethylene separator filled with 1.0 mol L^−1^ LiPF_6_/EC + DMC + EMC (30:40:30 vol%) electrolyte. The Li/NCM and Li/graphite cells underwent charging and discharging processes within voltage ranges of 2.5–4.2 V and 0.01–1.5 V, respectively, at a constant current of C/10 for 2 cycles, all at 20 °C.

The deactivation behaviors of the pouch cells using RSs were evaluated. The NCM electrode (area: 10 cm^2^) and the graphite electrode (area: 10.5 cm^2^) were positioned facing each other with the polyethylene separator in between and pouched in an aluminum laminate film bag. In the Ar-filled glovebox, the pouch cell was filled with 0.4–0.5 mL of 1.0 mol L^−1^ LiPF_6_/EC + DMC + EMC (30:40:30 vol%) and sealed. The pouch cell was charged and discharged for two cycles in the voltage range of 3.0–4.1 V at a constant current of C/10 or C/20, followed by full charging to 4.1 V at a rate of C/10.

The fully charged cell was opened in an Ar-filled glove box, and the RS solution, in which an RS was dissolved at 0.05 mol L^−1^ in a mixture of EC + DMC + EMC (30:40:30 vol%), was injected into the cell. After injection, the opened cell was sealed again. The injection amount of the RS solution was 1.268 mL, which corresponds to 10% of the cell capacity (17 mAh, as described in “Results and discussion”) under the assumption that the number of transferred electrons is 1. Thereafter, the cell was taken out of the glove box, and the OCV was monitored in a constant-temperature incubator at 20 °C. After the OCV showed no noticeable change, the cell was disassembled, and the NCM and graphite electrodes were washed with DMC. Thereafter, the electrode potentials versus Li were measured in the electrolyte solution of 1.0 mol L^−1^ LiPF_6_/EC + DMC + EMC (30:40:30 vol%).

### Deactivation of LIBs with Li deposition

The deactivation by the RSs was evaluated using LIBs with deposited Li. Two pouch cells were fabricated and conditioned to SOC100% under the same condition as above. Afterward, to deposit Li on the negative electrode surface, the cells were charged and discharged in the voltage range of 3.0–4.1 V at 20 °C, at a constant current of 4.5C for 20 cycles and subsequently at a constant current of 5.5C for 20 cycles. After the charge/discharge process, one cell was disassembled in an Ar-filled glove box to examine the state of Li deposition on the negative electrode surface. The other cell was subjected to deactivation using an RS agent (phenothiazine solution) under identical conditions as described above. After the cell voltage exhibited no discernable changes, it was disassembled within an argon-filled glove box to inspect the negative electrode surface.

### Supplementary Information

Supplementary Information.

## Data Availability

Raw data supporting the findings of this study are available from the corresponding author on reasonable request.
