# Supplementary material for: Battery deactivation with redox shuttles for safe and efficient recycling
Source: Sci Rep. 2024 Feb 11;14:3448. doi: 10.1038/s41598-024-53895-3 (PMC10859364; doi:10.1038/s41598-024-53895-3)
Supplement: Supplementary file 1 — Supplementary Information. [file 41598_2024_53895_MOESM1_ESM.pdf]

Supplementary Information

Battery deactivation with redox shuttles for safe and efficient recycling

Riho Mikita*, Akitoshi Suzumura, and Hiroki Kondo

Toyota Central R&D Laboratories, Inc., Nagakute, Aichi 480-1192, Japan

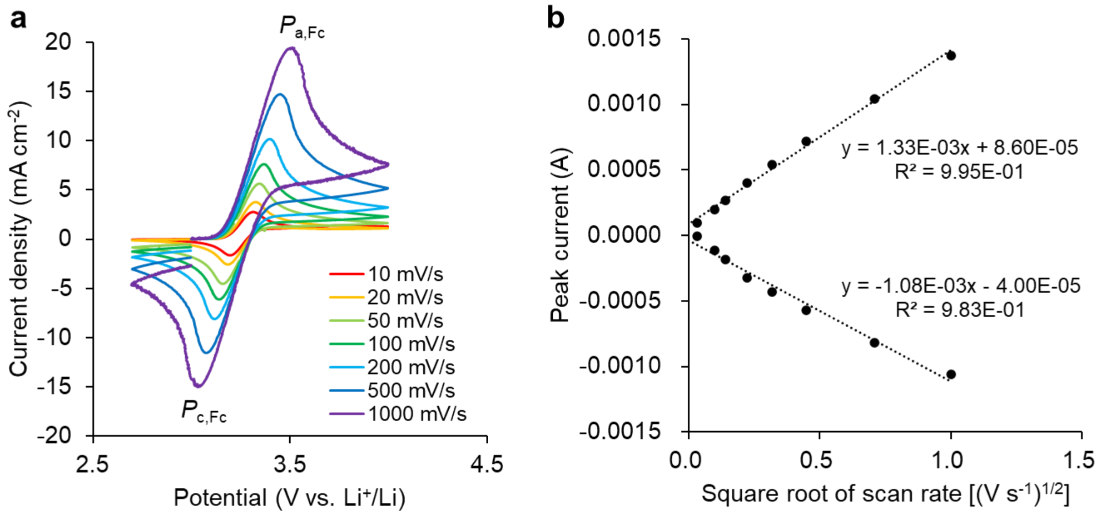

Figure S1. (a) Cyclic voltammograms of ferrocene with 1 M $LiPF_6$ as the supporting electrolyte in a mixture of ethylene carbonate (EC), dimethyl carbonate (DMC), and ethyl methyl carbonate (EMC) (volume ratio = 30:40:30) on a glassy carbon electrode at scan rates of 10, 20, 50, 100, 200, 500, and 1000 mV s^{-1}. All measurements were carried out at 20°C. (b) Plot of the peak current versus the square root of the scan rate.

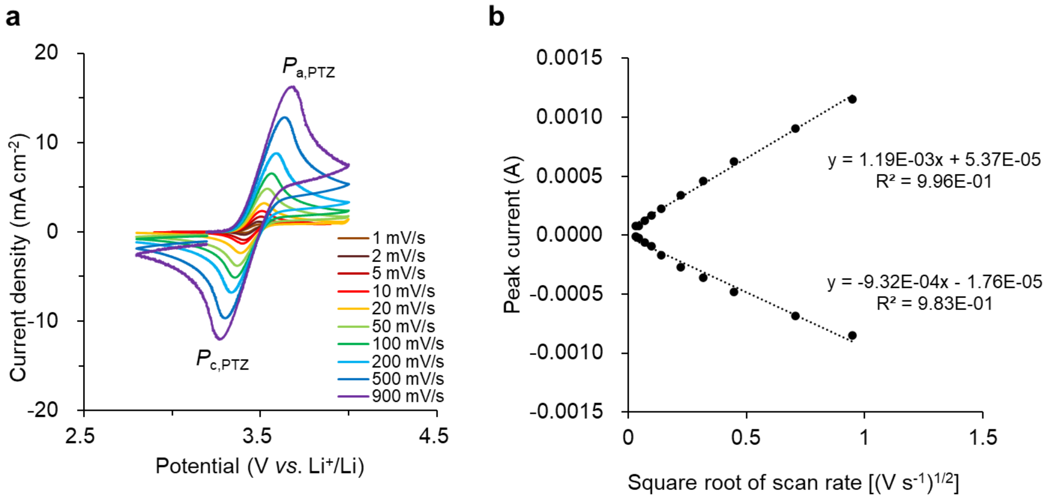

Figure S2. (a) Cyclic voltammograms of phenothiazine with 1 M $LiPF_6$ as the supporting electrolyte in a mixture of EC, DMC, and EMC (volume ratio = 30:40:30) on a glassy carbon electrode at scan rates of 1, 2, 5, 10, 20, 50, 100, 200, 500, and 900 mV s^{-1}. All measurements were carried out at 20°C. (b) Plot of the peak current versus the square root of the scan rate.

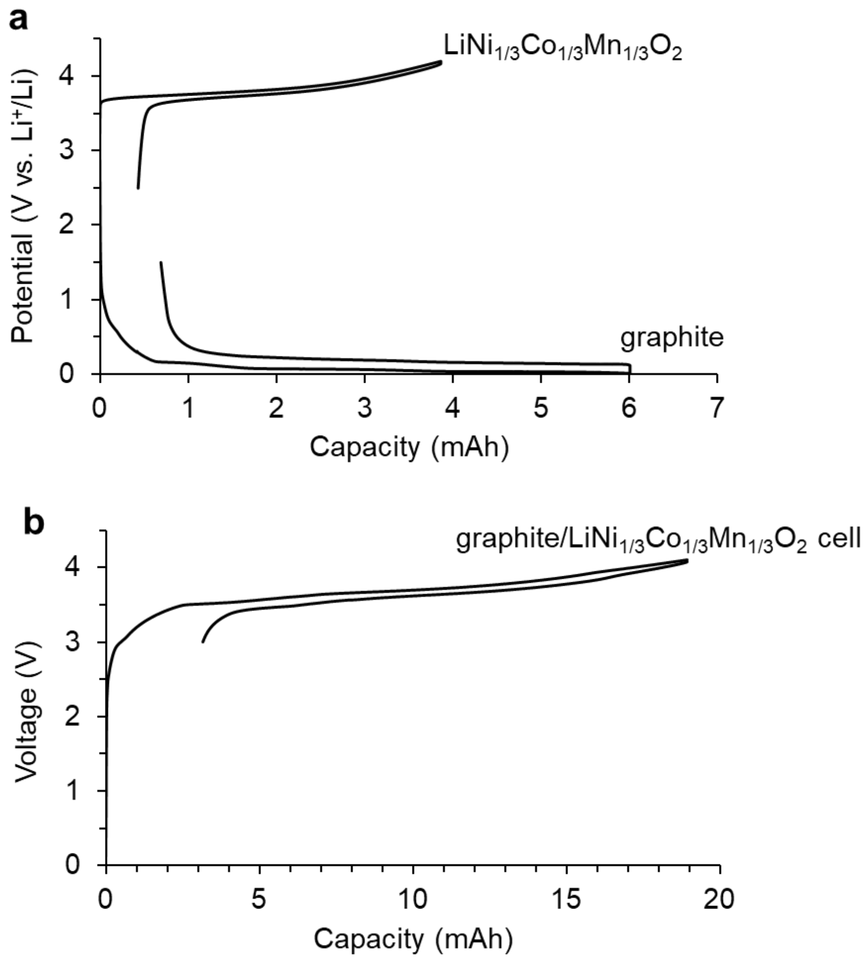

Figure S3. (a) The first charge/discharge curves of $LiNi_{1/3}Co_{1/3}Mn_{1/3}O_2$ and graphite electrodes. The area of the electrode used in the test is 2 cm^2. (b) The first charge/discharge curves of the graphite/$LiNi_{1/3}Co_{1/3}Mn_{1/3}O_2$ cell. The electrode areas of the graphite and $LiNi_{1/3}Co_{1/3}Mn_{1/3}O_2$ are 10.5 cm^2 and 10 cm^2, respectively.

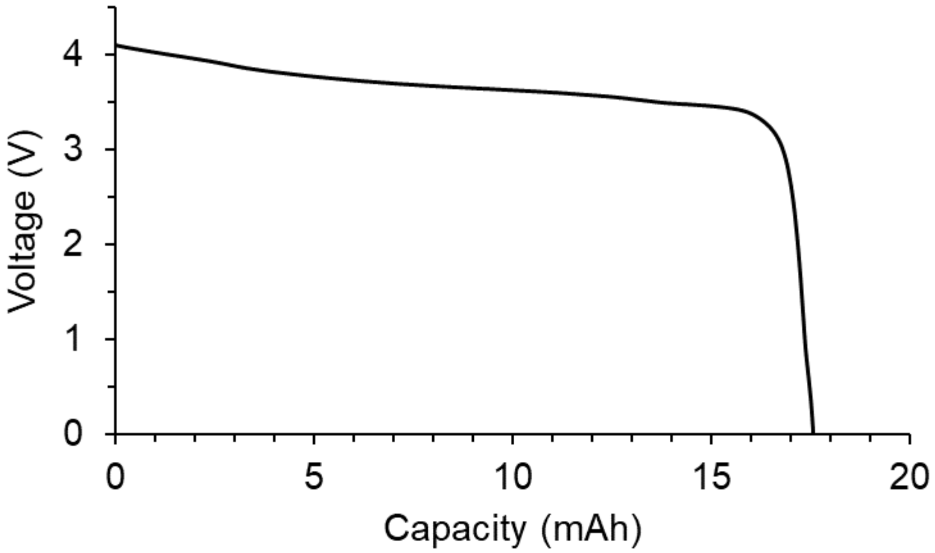

Figure S4. Voltage profile of the pouch graphite/$LiNi_{1/3}Co_{1/3}Mn_{1/3}O_2$ cell discharged to 0 V at a C/10 rate.

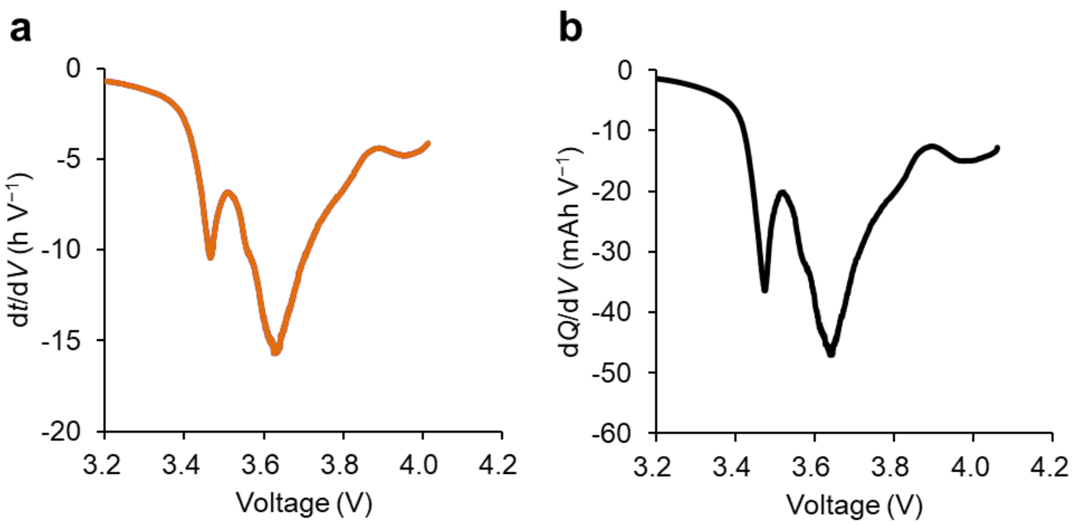

Figure S5. (a) Differential curve of time over the cell voltage (d*t*/d*V* curve) for the discharge curve with ferrocene. (b) Differential curve of the cell capacity over the cell voltage (d*Q*/d*V* curve) for the constant-current discharge curve.

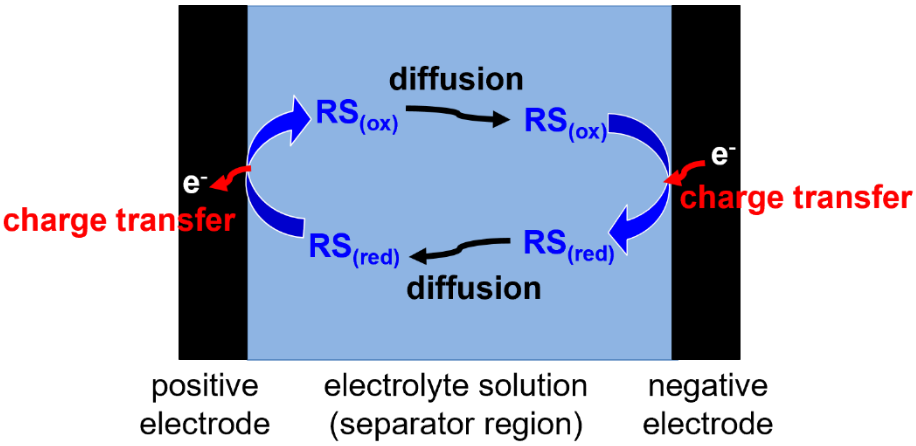

Figure S6. Schematic of LIB discharge with redox shuttles, which is assumed to be divided into two processes: charge transfer at the electrode/electrolyte interface and diffusion of the redox shuttle between the positive and negative electrodes.

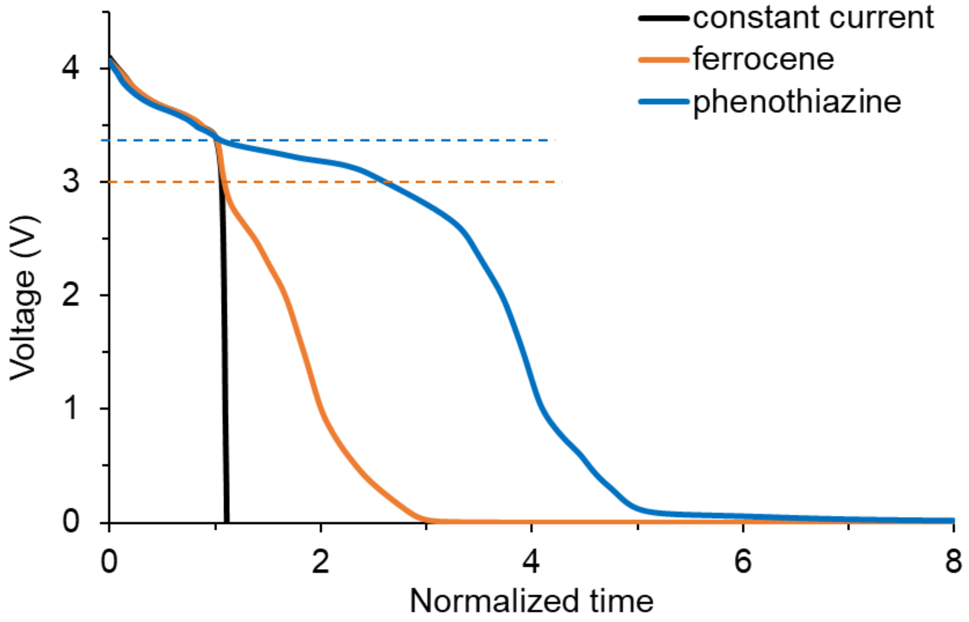

Figure S7. Comparison curve of the discharge between adding a solution of ferrocene or phenothiazine. The horizontal axis is normalized by time at a voltage of 3.4 V. The orange and blue broken lines show the voltage at which the discharge rate decreased when ferrocene and phenothiazine were used as deactivating agents, respectively.

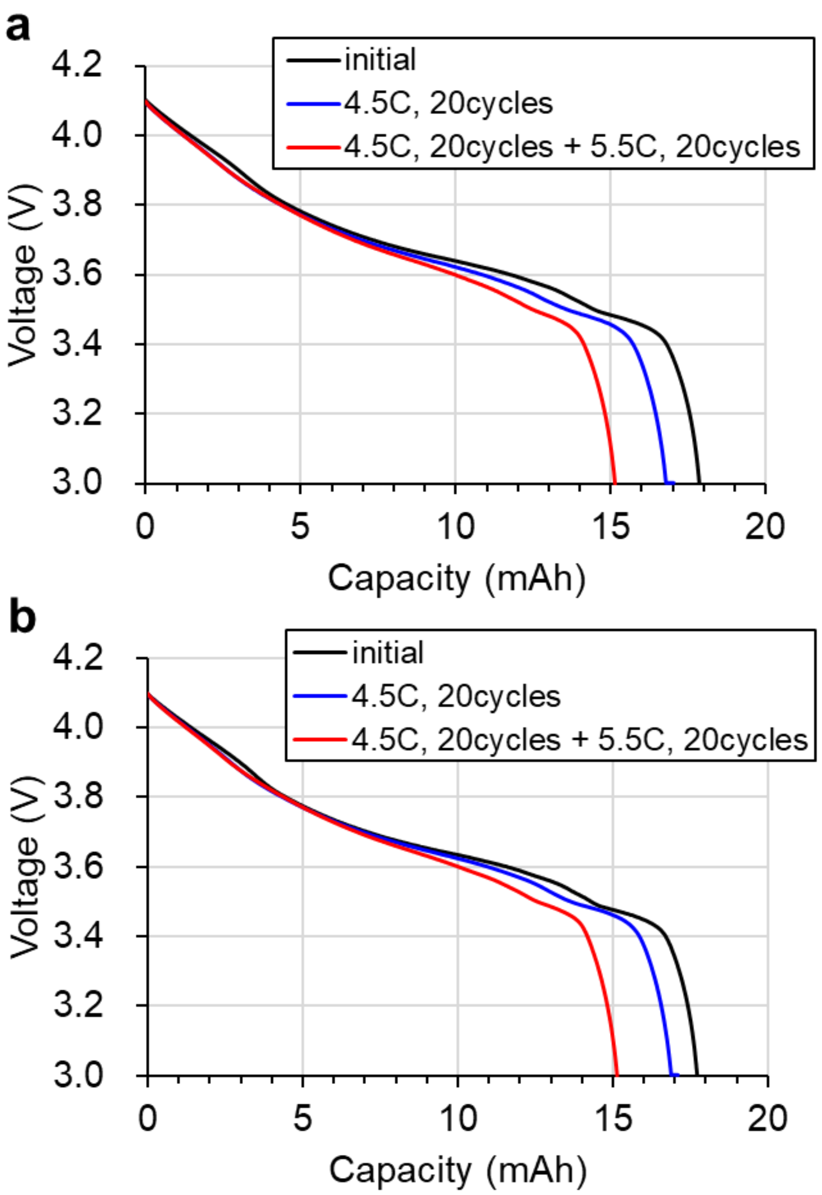

Figure S8. Initial discharge curve and curves after high-rate charge–discharge cycles for two pouch cells used for the present experiment. The cell for panel (a) was conditioned to SOC100% after the high-rate cycles and disassembled in an Ar-filled glove box to examine the deposited Li on the negative electrode, whereas the cell for panel (b) was conditioned to SOC100% and subsequently deactivated with the phenothiazine solution.

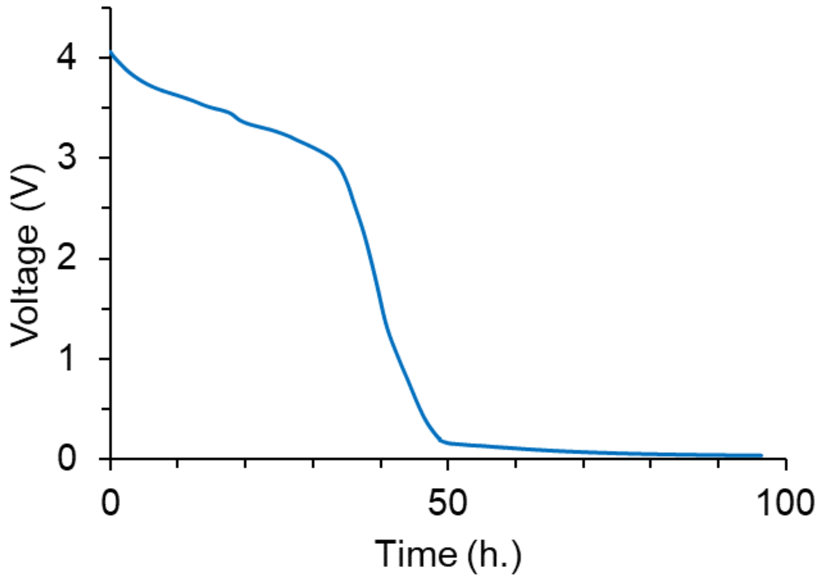

Figure S9. Discharge curve of the graphite/$LiNi_{1/3}Co_{1/3}Mn_{1/3}O_2$ cell with Li deposited on the negative electrode surface by adding phenothiazine solution.

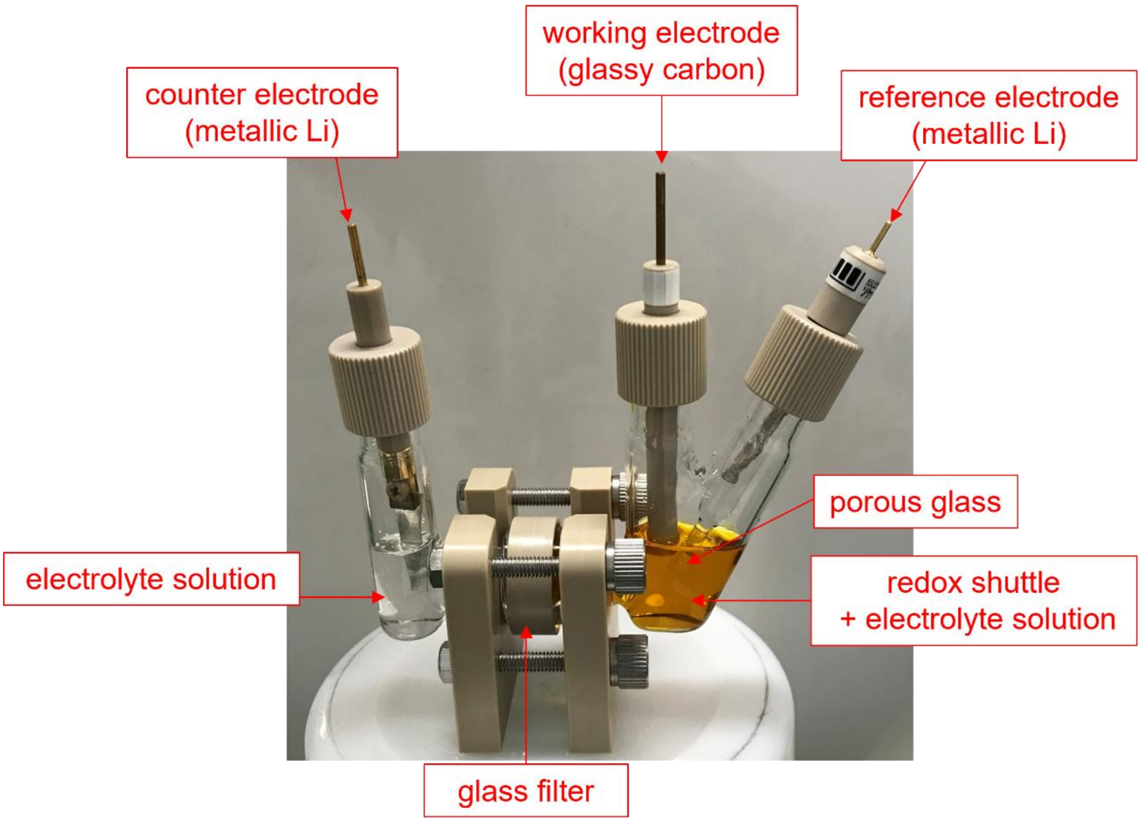

Figure S10. Image of H-type cells with three electrodes used in the cyclic voltammetry measurements of the redox shuttles. The counter and reference Li electrodes were separated from the redox shuttles through a glass filter and porous glass, respectively.

Table S1. Parameters used for calculating the diffusion coefficients of the redox shuttles.

Parameter	Brief explanation	Value
n_a, n_c	Number transferred electrons	1
F	Faraday's constant	96,485 (C mol^{-1})
A	Electrode area	7.07×10^{-6} (m^2)
α_a, α_c	Electron-transfer coefficient	0.5
R	Gas constant	8.314 (J K^{-1} mol^{-1})
T	Temperature	293.15 (K)
c^*_R, c^*_O	Concentration of the redox shuttle	50 (mol m^{-3})

Table S2. Absolute values of the slope of the fitting lines for the plot of the peak current versus the square root of the scan rate and the obtained diffusion coefficients of the redox shuttles.

	Absolute value of the slope	Diffusion coefficient ($m^2 s^{-1}$)
Ferrocene	1.33×10^{-3}	3.1×10^{-10}
	1.08×10^{-3}	2.1×10^{-10}
Phenothiazine	1.19×10^{-3}	2.5×10^{-10}
	9.32×10^{-4}	1.5×10^{-10}
